# A Fractal Nature for Polymerized Laminin

**DOI:** 10.1371/journal.pone.0109388

**Published:** 2014-10-08

**Authors:** Camila Hochman-Mendez, Marco Cantini, David Moratal, Manuel Salmeron-Sanchez, Tatiana Coelho-Sampaio

**Affiliations:** 1 Institute of Biomedical Sciences, Federal University of Rio de Janeiro, Rio de Janeiro, Brazil; 2 Institute of Biophysics Carlos Chagas Filho, Federal University of Rio de Janeiro, Rio de Janeiro, Brazil; 3 Division of Biomedical Engineering, School of Engineering, University of Glasgow, Glasgow, United Kingdom; 4 Center for Biomaterials and Tissue Engineering, Universitat Politècnica de València, València, Spain; University of Quebec at Trois-Rivieres, Canada

## Abstract

Polylaminin (polyLM) is a non-covalent acid-induced nano- and micro-structured polymer of the protein laminin displaying distinguished biological properties. Polylaminin stimulates neuritogenesis beyond the levels achieved by ordinary laminin and has been shown to promote axonal regeneration in animal models of spinal cord injury. Here we used confocal fluorescence microscopy (CFM), scanning electron microscopy (SEM) and atomic force microscopy (AFM) to characterize its three-dimensional structure. Renderization of confocal optical slices of immunostained polyLM revealed the aspect of a loose flocculated meshwork, which was homogeneously stained by the antibody. On the other hand, an ordinary matrix obtained upon adsorption of laminin in neutral pH (LM) was constituted of bulky protein aggregates whose interior was not accessible to the same anti-laminin antibody. SEM and AFM analyses revealed that the seed unit of polyLM was a flat polygon formed in solution whereas the seed structure of LM was highly heterogeneous, intercalating rod-like, spherical and thin spread lamellar deposits. As polyLM was visualized at progressively increasing magnifications, we observed that the morphology of the polymer was alike independently of the magnification used for the observation. A search for the Hausdorff dimension in images of the two matrices showed that polyLM, but not LM, presented fractal dimensions of 1.55, 1.62 and 1.70 after 1, 8 and 12 hours of adsorption, respectively. Data in the present work suggest that the intrinsic fractal nature of polymerized laminin can be the structural basis for the fractal-like organization of basement membranes in the neurogenic niches of the central nervous system.

## Introduction

Laminin is the major molecular component of the basement membrane, a specialized type of extracellular matrix characterized by a flat sheet-like geometry. Laminin regulates a variety of biological phenomena, including the provision of boundaries between neighboring tissues, the establishment of molecular filters and the modulation of cell behavior [1]-[3]. The quaternary structure of laminin is given by three different polypeptide chains, which associate to form a cross-shaped heterotrimer, exhibiting one long and three shorter arms. In the three-dimensional space laminin has the shape of a three-leafed clover, in which the leaves correspond to the three short arms while the stem corresponds to the long arm. This 3-D structure is particularly adequate to favor the assembly of a sheet-like polymer, where the three short arms simultaneously interact with each other within a single spatial plane, while the long arm is left available to interact with the surface of contiguous cells [4].

As a consequence of its structural properties, laminin can spontaneously self-polymerize in a test tube, requiring either a minimal protein concentration [5] or a decrease in the solution pH [6], [7]. Polymers formed upon pH acidification, designated as polylaminin (polyLM^1^), present specific signaling properties and have been shown to stimulate the outgrowth of neurites with at least twice the efficiency of ordinary laminin (LM), namely a matrix obtained by adsorbing the protein diluted in neutral pH onto a glass coverslip [8]. PolyLM was also shown to reverse the loss of migratory and neuritogenic potentials of cortical neurons and to promote the survival and the proliferation of axotomized retinal ganglion cells, both isolated from newborn rodents [9]. Finally, it was demonstrated that polyLM, but not the laminin protein diluted in neutral buffer, promoted axonal regeneration and functional recovery after spinal cord injury in rats [10].

The morphology of polyLM has previously been studied both at the micro and at the nanometer scales. Using negative staining followed by transmission electron microscopy it was possible to characterize it as a regular polygonal network displaying the same features of the natural laminin networks assembled by living cells [11], [12]. In such polymers the unit polygon was a hexagon of approximately 30 nm of side, which well corresponded to the size of each short arm in the laminin molecule. Curiously, a polygonal array of comparable features was observed at a three orders of magnitude larger scale when immunolabeled polyLM was analyzed by fluorescence microscopy [7], [8], [13].

The fact that polyLM observed at different magnifications showed similar structures suggested that the polymer could be a fractal structure. Nevertheless, the lack of images obtained at intermediary magnifications, as well as the insufficient resolution of previous epifluorescence photomicrographs, prevented the search for a fractal dimension of polyLM. In the present work we used confocal fluorescence microscopy (CFM), scanning electron microscopy (SEM) and atomic force microscopy (AFM) to obtain a detailed structural characterization of polyLM that would permit assessment of its putative fractal nature. We showed that polyLM presented a fractal dimension, which increased its complexity upon accumulation of the polymer on a flat substrate. These findings may have important implications as they can provide an intrinsic molecular basis for the fractal-like organization of the basement membranes present at the neurogenic niches in the adult central nervous system.

## Experimental Procedures

### Preparation of laminin matrices

PolyLM was produced by diluting EHS laminin (laminin 111; Invitrogen) to 50 µg/mL with 20 mM sodium acetate (pH 4), containing 1 mM CaCl_2_. The laminin protein has previously been shown to polymerize in solution within a few minutes after dilution in acidic buffer, independently of its concentration [6]. The polymers produced in solution (polyLM) were adsorbed to glass coverslips to produce the matrices used here for microscopic analyses. LM was produced by diluting EHS laminin to 50 µg/mL with 20 mM Tris-HCl (pH 7) containing 1 mM CaCl_2_. This concentration was below the critical protein concentration necessary to trigger laminin polymerization in solution at neutral pH [5], so that the LM matrix was formed as the protein decanted and raised its concentration at the glass surface. In order to avoid unwanted polymerization in the highly concentrated stock solution of EHS laminin (0.5–1.5 mg/mL), working aliquots (2–10 µL) were stored frozen and individually thawed in ice immediately before dilution. Unless otherwise indicated, incubations with glass coverslips were carried out at 37°C for 12 hours, which is known to be sufficient to warrant that at least 60% of the protein would decant and adsorb regardless of the solution pH [8].

### Immunostaining and confocal microscopy

Laminin matrices adsorbed on glass coverslips were fixed with paraformadehyde 4% for 20 min and prepared for indirect immunofluorescence analysis. Coverslips were washed 3 times for 5 min in PBS and incubated with bovine serum albumin 5% in PBS (PBS-BSA 5%) for 30 min. The primary antibody was a polyclonal rabbit anti-laminin antibody (1∶30, Sigma-Aldrich, no. L9393). After overnight incubation at 4°C, coverslips were washed 3 times for 5 min in PBS and incubated with an Alexa Fluor 488 anti-rabbit secondary antibody (1∶300; Life Technology, no. A-11001) for 1 hour at room temperature. They received three 5-min washes with PBS and one with distilled water before being mounted in n-propyl gallate in 80% glycerol (Sigma-Aldrich). Confocal images were obtained in a Leica TCS-SP5 confocal laser scanning microscope using a HCX PL APO lambda blue 63X objective for oil immersion (1.4 of numerical aperture). Images correspond to renderized stacks of 74 optical slices obtained with a zoom of 3.2 at each 21.4 µm (total width of 1530.8 µm).

### Scanning electron microscopy

Laminin matrices attached to coverslips were fixed in Karnowsky reagent (4% PA and 0.5% glutaraldehyde in 0.1 M cacodylate buffer, pH 7.2) for 2 hours, washed three times with sodium cacodylate buffer 0.1 M, pH 7.2, dehydrated through increasing concentrations of ethanol and dried in E300 (Polaron, Quorum Technologies Ltd, Laughton, United Kingdom) critical point. The samples were then coated with a thin layer of gold sputter (Leica EM MED020) and viewed under a scanning electron microscope Jeol JSM6300.

### Transmission electron microscopy

Transmission electron microscopy after negative staining was carried out as previously described [11]. Briefly, 5 µl of laminin in acidic buffer (polyLM) was deposited on a Formvar-coated copper grid and a 5 µl drop of 2% uranyl acetate was added over it. Samples were visualized in a Zeiss EM 900 transmission electron microscope operated at 80 kV.

### Atomic force microscopy

Laminin matrices were fixed in 4% paraformaldehyde for 20 minutes and dried in critical point dryer E300 (Polaron, Quorum Technologies Ltd, Laughton, United Kingdom). AFM analyses were performed using a Multimode AFM equipped with a NanoScope IIIa controller (Bruker) operating in tapping mode in air; the Nanoscope 5.30r2 software version was used for image processing and analysis. Si-cantilevers MPP-21120 from Bruker were used, with force constant of 3 N.m^−1^ and resonance frequency of 75 kHz. The phase signal was set to zero at a frequency 5–10% lower than the resonance one. Drive amplitude was 200 mV and the amplitude set-point (A_sp_) was 1.4 V. The ratio between the amplitude set-point and the free amplitude (A_sp_/A_0_) was kept equal to 0.7.

### Quantification of laminin adsorption

The amount of adsorbed laminin was measured using a Micro BCA Protein Assay Kit (23235#, Thermo Scientific Pierce). Following the standard protocol, the working reagent (WR) was prepared from 25 parts of MA (sodium carbonate, sodium bicarbonate and sodium tartrate in 0.2 N NaOH), 24 parts of MB (4% BCA in water) and 1 part of MC (4% cupric sulfate, pentahydrate in water). As standards, nine bovine serum albumin (BSA) solutions with concentrations ranging from 0.0 to 200 µg/mL were prepared by dissolving BSA in the buffers used for laminin adsorption (acidic buffer and neutral buffer). The amount of adsorbed protein was calculated by measuring the amount of protein remaining in the supernatant at each time point. The samples and the standards were incubated with WR 1∶1 at 37°C for two hours before cooling to room temperature. Then, the absorbance at 562 nm was measured with the plate reader Victor3 (PerkinElmer, Waltham, Massachusetts). All the absorbance values were corrected by the average 562 nm absorbance reading of the blank standard replicates. Each measurement was performed in duplicate.

### Calculus of the fractal or the Hausdorff dimension

All image processing and analysis was done using an in-house software developed under MATLAB R2006a (The MathWorks, Inc., Natick, MA). The fractal dimension was determined using a box-counting dimension estimate of the Hausdorff dimension, which is a descriptor of the complexity of geometry of a given set [14]. As the definition of the Hausdorff dimension does not offer any guideline to an estimate calculation, the box-counting dimension has been used. The box-counting dimension is an estimate of the Hausdorff dimension based on covering the investigated set with a fixed grid of size *r* [15], [16]. This box-counting dimension can be calculated using the equation (1):
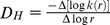
(1)where *k*(*r*) is a number of grid boxes that contain any part of the investigated set, and where the process is repeated with different values of the grid size. *D_H_* is known as the Hausdorff dimension, the Minkowski-Bouligand dimension, the Kolmogorov capacity or dimension, or simply the box-counting dimension.

Before applying the box-counting algorithm, the grayscale image histogram was equalized and the resulting image was binarized using the Otsu's method [17]. Finally, the box-counting estimate was calculated on this image. All this process was performed on ten different image crops of the same size (100 pixels×100 pixels) of the original images, and all these image crops were rescaled ten times to obtain an image big enough to apply the box-counting algorithm.

## Results

### Three-dimensional structures of polyLM and LM assessed by confocal fluorescence and scanning electron microscopy

**Figure 1:**
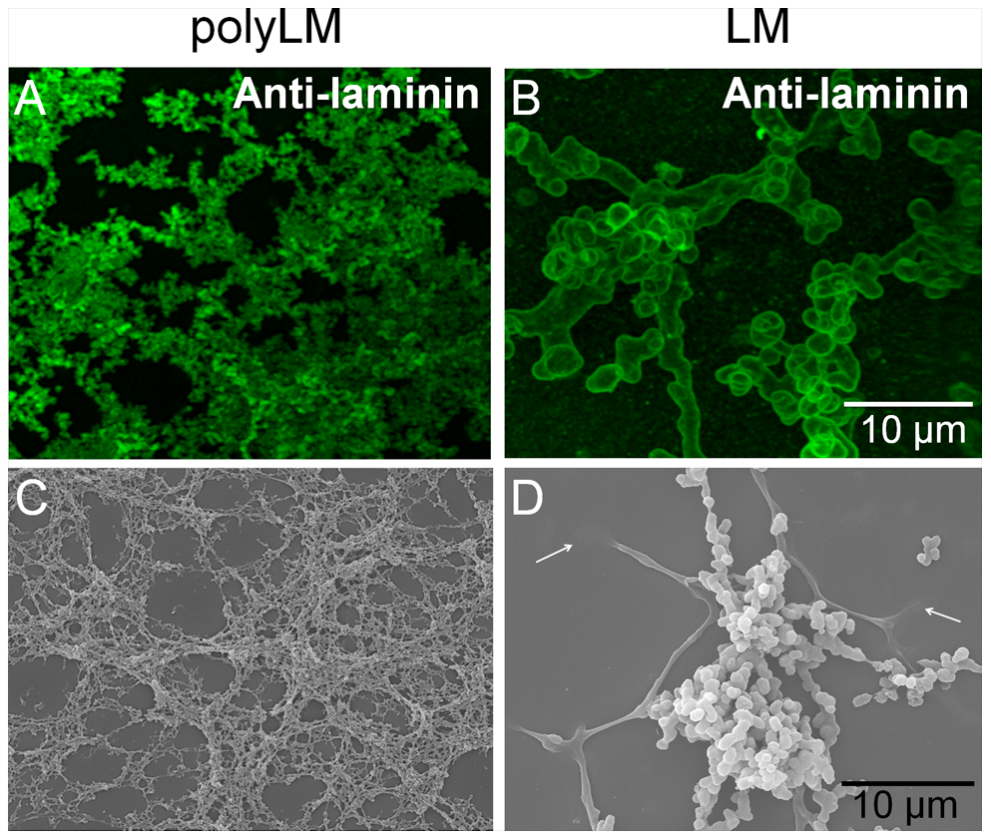
Three-dimensional structure of laminin polymers under confocal fluorescence microscopy and scanning electron microscopy. Laminin was incubated on glass coverslip for 12 hours in acidic (polyLM) or in neutral buffer (LM). (A, B) Indirect immunofluorescence was performed using a polyclonal antibody against laminin. The images depict z-stacks obtained by the superposition of 74 confocal slices renderized using the software 7.2.3 (Bit-plane; free trial). (C, D). Scanning electron micrographies (SEM) of the polymers shown at a similar magnification. Arrows in D point to lamellar deposits of laminin. The scale bars apply to panels A–D and represent 10 µm.

Matrices of adsorbed laminin obtained at acidic (polyLM) or neutral pH (LM) were initially analyzed by reconstructing a series of 74 confocal optical slices renderized to reveal their 3-D structure. PolyLM corresponded to a sponge-like network of an apparently homogeneous density (Fig. 1A). Labeling was equally distributed, indicating that the antibody could evenly access protein epitopes within the polymer. On the other hand, LM presented a branched morphology, resembling that of a marine coral, which protruded from the glass surface (Fig. 1B). The antibody did not penetrate the spherical protein clumps and only their contours were brightly stained. Noteworthy was the observation that in LM a significant amount of protein adsorbed directly to the glass coverslip. This likely corresponds to the protein not incorporated to the aggregates. As a comparison, the background of the image depicting polyLM was dark, indicating that virtually all laminin protein engaged into that polymer. The 3-D structures of the two laminin matrices can be better appreciated in the animation movies presented as Movies S1 and S2.

The 3-D structures of polyLM and LM were additionally investigated by using SEM. While polyLM was again seen as a homogeneous mesh, LM showed at least two structural components (Fig. 1C and D). Besides the spherical aggregates already identified by CFM, it was possible to devise the presence of rod-like structures. In addition, the tips of these rods possessed lamellar terminations, suggesting the occurrence a third structural component of the LM matrix (arrows in Fig. 1D).

### Kinetics of formation of laminin matrices

**Figure 2:**
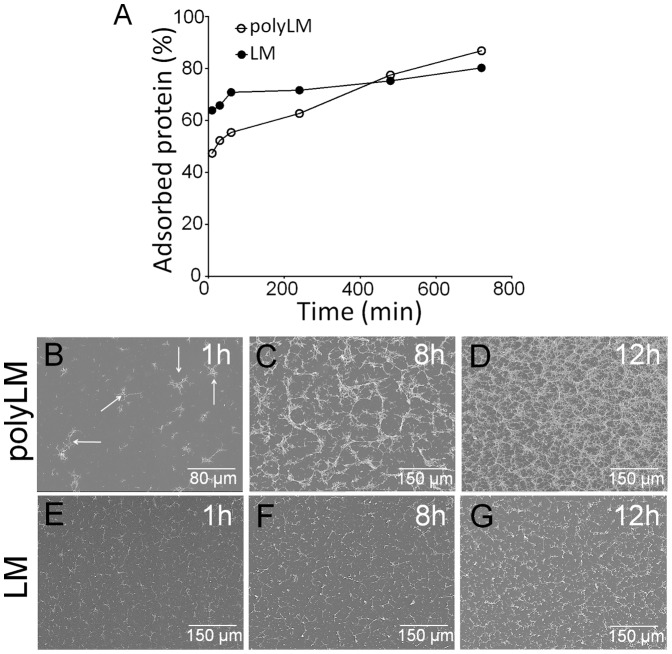
Kinetics of adsorption of polyLM and LM. (A) Laminin was incubated in acidic (polyLM) or neutral buffer (LM) and a kinetic of adsorption was carried out by collecting aliquots of the supernatant at 10 minutes, 30 minutes, 1 hour, 4 hours, 8 hours and 12 hours for quantification of the protein content remaining in solution. Open symbols represent polyLM and closed symbols represent the LM. (B–G) SEM images show the polymers obtained in acidic (B–D) or neutral (E–G) buffers at the indicated times. The arrows (B) point to structured polymers observed at 1 hour of incubation.

The amount of adsorbed laminin was calculated by measuring the concentration of protein remaining in solution at 20, 40 and 60 min and after 4, 8 and 12 hours of adsorption (Fig. 2A). Laminin decanted more quickly at neutral pH to form the LM matrix. More than 60% of the protein was absorbed after 20 min of incubation and such amount slightly increased up to 80% at 12 hours. On the other hand, at acidic pH the kinetic of adsorption was more linear, whereas 45% of the protein was adsorbed at 20 min and such proportion increased to 90% at 12 hours. The morphologies of the two matrices were analyzed at 1, 8 and 12 hours by SEM (Fig. 2B–G). Already at 1 hour polyLM decanted exhibiting a structured 2-D morphology consistent with the formation of the meshwork observed at 12 hours (Fig. 2B, D).

### Characterization of the seed units of polyLM and LM

**Figure 3:**
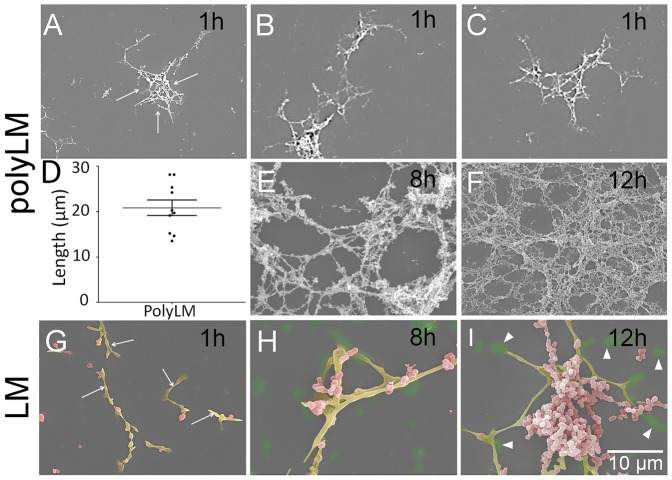
Characterization of polymer units in polyLM and LM. Laminin polymers were analyzed at high magnification in order to characterize the morphologies of the seed units of each polymer. (A–C) At 1 hour polyLM forms star-like 2D structures as exemplified in the three panels. (D) The sizes of the longer axes in these structures were quantified and shown to average at 20.84±5.449 µm. (E, F) High magnification images of polyLM at 8 and 12 hours show a meshwork pattern compatible with the deposition of the star-like structures. (G–I) LM observed at high magnification reveals three types of seed structures: rods (pseudocolored yellow), spheres (pseudocolored pink) and lamellas (pesudocolored green). The scale bar in I applies to all panels and represents 10 µm.

When the seed unit of polyLM was observed at higher magnification one could see that it was itself composed of polygons in a planar organization. Such seed unit was consistently present in matrices obtained within one hour of incubation (Fig. 3A–C). The extent of their longest axes ranged between 14 and 28 µm (Fig. 3D). After 8 and 12 hours of incubation we could already observe the presence of a mesh-like network whose morphology was compatible with the overlay of the seed units observed at one hour; these units however could no longer be distinguished within the meshwork (Fig. 3E, F). In LM we could observe lamellar-like deposits adsorbed directly to the coverslips (pseudo colored green in Fig. 3). These lamellar deposits were either located at the end of rod-like structures (yellow) or they appeared as individual patches (arrowheads in Fig. 3I). The seed unit was pseudo colored as to reveal the three types of deposits seen in LM, namely spheres (pink), rods (yellow) and lamellar deposits (green) (Fig. 3G–I).

### Three-dimensional structures of polyLM and LM assessed by atomic force microscopy

**Figure 4:**
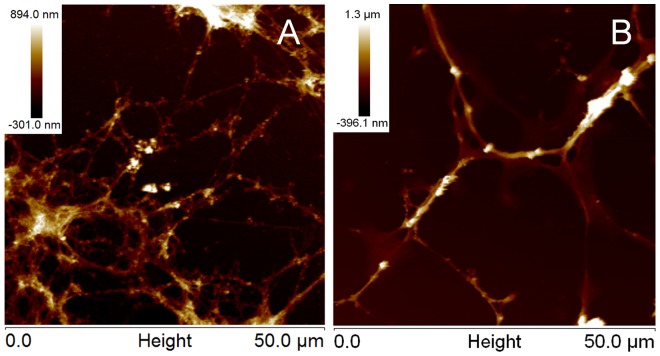
Overall morphology of polyLM and LM under AFM. Atomic force microscopy images of polyLM (A) and LM (B) are shown in height mode after critical-point drying of the samples. Both matrices were obtained by incubating laminin with glass coverslips in the appropriate buffers for 12 hours. The scanned area was 2500 µm2.

We next examined the 3-D features of polyLM and LM using AFM. When areas of 50×50 µm were scanned, the overall appearances of polyLM and LM were comparable to those visualized by CFM and SEM (Fig. 1). PolyLM displayed the morphology of a multilayered meshwork containing homogeneous struts (Fig. 4A), while LM exhibited rods and lamellar deposits (Fig. 4B). The spherical aggregates previously seen under confocal fluorescence (Fig 1B) and SEM (Fig 1D and 3G–I) were not observed under AFM due to their large size, which was beyond the Z scan range of the AFM.

**Figure 5:**
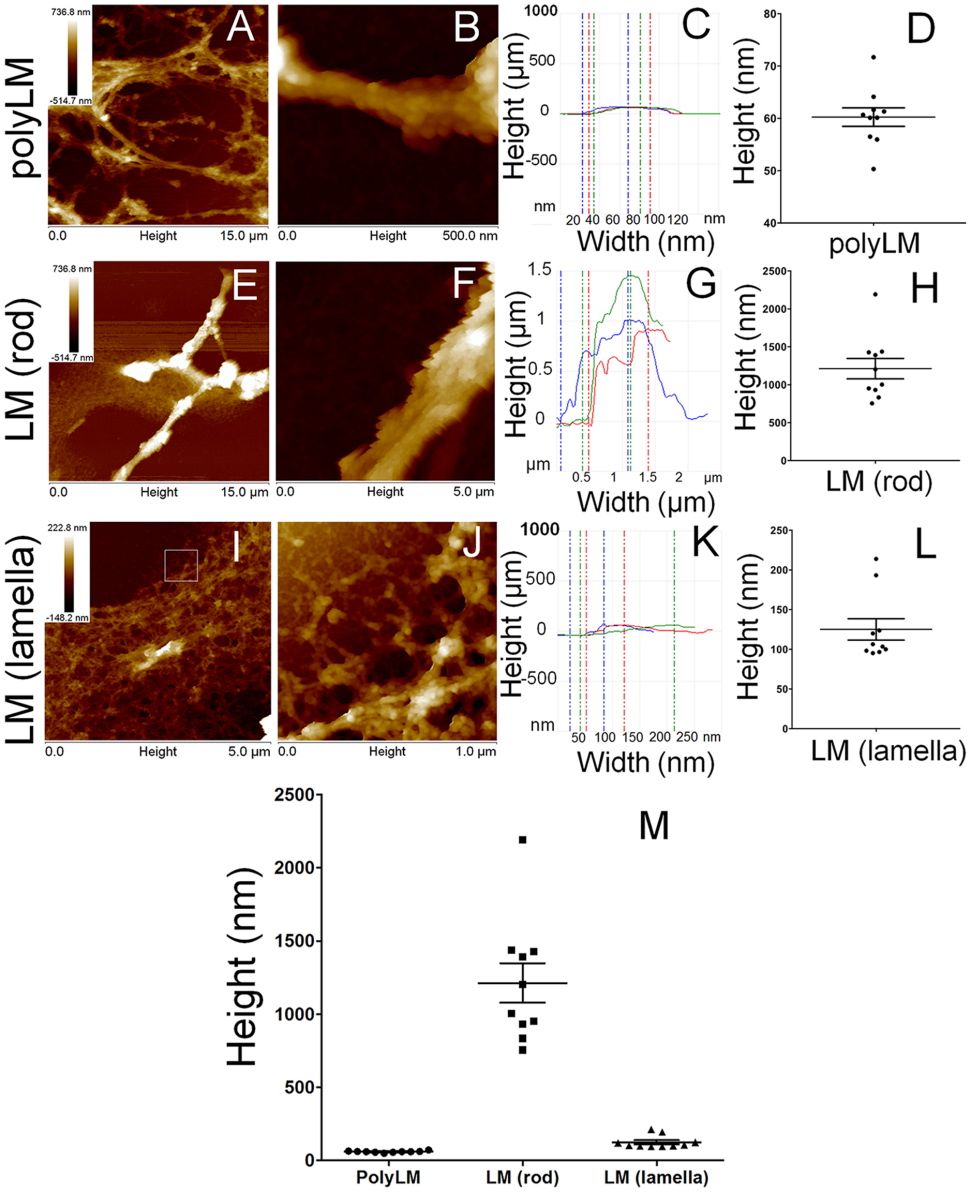
AFM analysis of polyLM and LM at increasing magnifications. PolyLM (A, B) and LM (E, F, I, J) obtained as described in Figure 4 were scanned in areas of 225 µm2 (A, E, I) or 0.25 µm2 (B, F, J) and shown in height mode. In order to determine the thickness of the structural units forming each polymer, the heights of 10 struts were calculated in the fields depicted in B (struts of the polyLM mesh), F (rods in LM) and J (lamellas in LM). Considering that both matrices were multilayered, each structure selected for measurement followed the criteria of being the closest possible to the support (glass coverslip). Panels C, G and K depict examples of three measurements and panels D, H and L show the distribution of the values obtained for each 10 structures. The white square in I represents an area at the edge of the lamellar structure used for the height measurement. Panel M shows the distribution of heights obtained at each condition all together for comparison.

To further characterize the homogeneity of the struts in polyLM, the matrix was scanned at higher magnifications (Fig. 5A, B). In fields of 0.5×0.5 µm, we measured the heights of 10 individual struts of the mesh, chosen as those in direct contact with the glass support, i.e., those in the bottom layer of the mesh. We found values ranging between 50 and 73 nm, with a mean height of 60.25±1.764 nm (Fig. 5C, D). In the heterogeneous LM matrix, we measured the heights of rods and lamellar deposits, which displayed average heights of 1213±134.6 nm (Fig. 5E–H) and 125.1±13.51 nm (Fig.5I–L), respectively.

### Evidence of a fractal nature for polyLM

**Figure 6:**
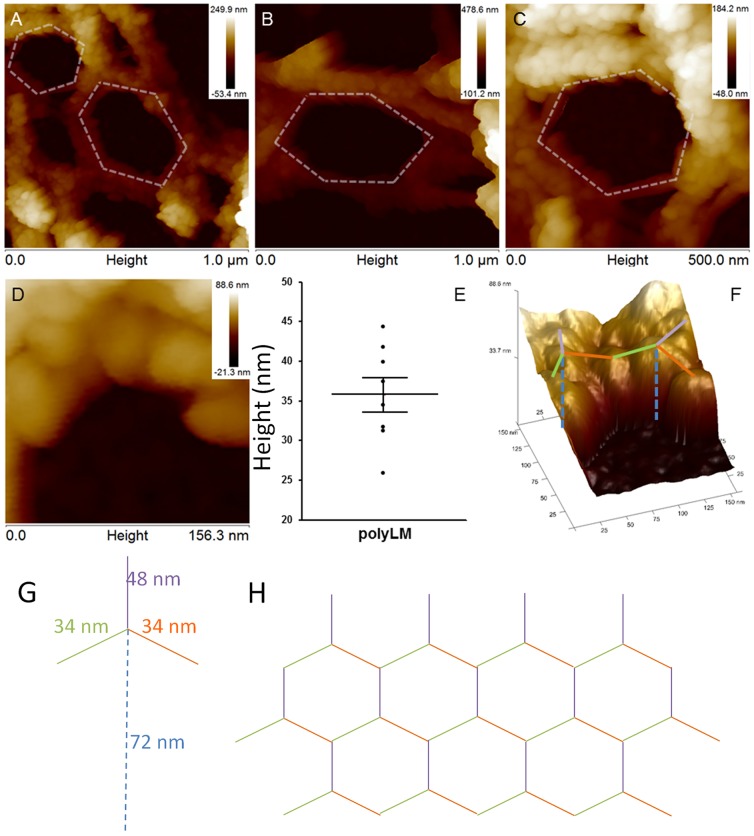
Atomic force microscopy reveals the occurrence of hexagonal-like figures in polyLM. AFM was performed on polyLM matrices obtained as described in Figure 4 and areas of 1 (A, B) or 0.25 µm^2^ (C) were scanned in height mode. Hexagons-like figures similar to those occurring in natural laminin polymers [12] were identified. These hexagons were visible at different magnifications (A–C) and presented variable side lengths (sketched with white dashed lines), but they were never as short as 30 nm as they should be to correspond to the short arm of a laminin molecule. The smallest distinguishable structures contained within the sides of the hexagons were little globules (D) whose size and spacing was measured in images of 0.02 µm^2^ (D). Panel E shows the distribution of spacing values, which are compatible with the characteristic length (∼30 nm) of laminin polymerized via the short arms. Panel F depicts a three-dimensional reconstruction of the same area shown in panel D, with superposition of compatible locations of laminin molecules. Schemes of one individual laminin molecule (long arm dashed and short arms colored blue, green and orange), with indication of its characteristic dimensions (G) and of the hexagonal polymer generated by the interaction between individual laminin molecules (H) are also shown.

When polyLM was observed at a higher magnification with AFM it was possible to observe the occurrence of figures that matched the hexagon-like shape of polymerized laminin at the molecular level (Fig. 6A–C). Such figures were still one order of magnitude larger than the basic hexagons formed by the association of the short arms of individual laminin molecules (Fig. 6G, H) [4], [12]. In that structure, each side of the hexagon possesses ∼30 nm, resulting from the interaction of the laminin short arms (35–50 nm long). The sides of the putative hexagons observed here were larger and their length was in the range of a few hundreds of nanometers. Nevertheless, these polygons were made out of small globules with a diameter and a spacing around 30–40 nm, compatible with the characteristic length of laminin polymerized through interaction between short arms (Fig. 6D–F). These structures could not be further resolved via AFM.

**Figure 7:**
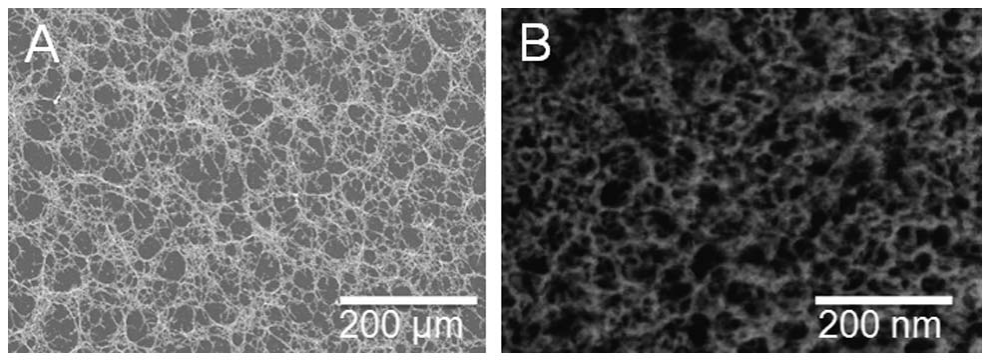
PolyLM displays similar morphologies at both 200 and 200,000 fold magnifications. Images of polyLM were obtained using SEM (A) or transmission electron microscopy (B) after negative staining. Under SEM the magnification was 200 fold while under TEM it was 200,000 fold. Note that the observed patterns were alike despite the 1000 fold increase in magnification.

The results obtained up this point suggested that the hexagonal network formed by the association of individual laminin molecules could reproduce itself at higher levels of organization. In order to investigate this hypothesis we compared images of polyLM obtained with SEM and with transmission electron microscopy after negative staining (Fig. 7). Surprisingly, the morphologies of polyLM were very similar under SEM (Fig. 7A) and transmission electron microscopy (Fig. 7B) regardless of a difference in magnification of 1,000 fold. This observation suggests that polyLM presents a fractal nature.

### Determination of the fractal dimension of polyLM

**Figure 8:**
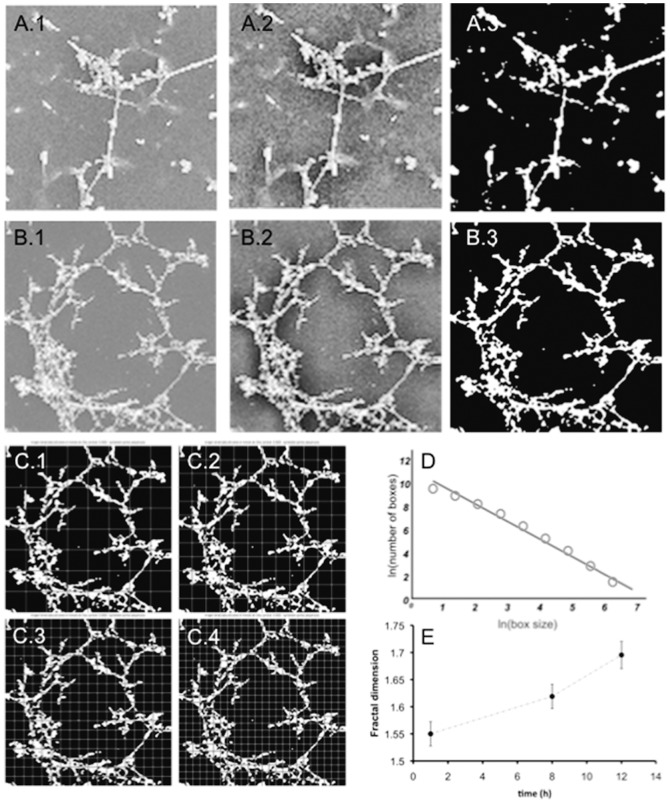
Calculation of the fractal dimension (Hausdorff estimate). (A, B) Image processing in order to prepare the image for the box-counting algorithm for LM (A) and polyLM (B). 1, original image; 2, original image in which the histogram has been equalized; 3, binarized image using Otsu's method). (C) From images A.3 and B.3 the Hausdorff dimension estimates can be calculated superimposing a grid of variable size (C.1-C.4, examples of the same image on which a grid of variable size has been superimposed). (D) Repeating the previous process for different values of grid size and computing the number of grid boxes that contain any part of the investigated set, the Hausdorff dimension or simply the box-counting dimension can be calculated. (E) Fractal dimension calculated for polyLM structures as a function of time.

Based on evidence that polyLM possessed a fractal structure, we analyzed images of polyLM obtained at increasing incubation times in search for its fractal dimension. Fractal or Hausdorff dimensions of increasing complexities were found for polyLM as the adsorption time increased from 1 to 12 h (Fig. 8). The calculated values were 1.55, 1.62 and 1.70 after 1, 8 and 12 hours of adsorption, respectively. By contrast, the LM matrix did not present a fractal structure from which a fractal dimension could be obtained.

## Discussion

In the present work we described that two matrices of laminin, polyLM and LM, obtained in different conditions presented highly different structures when observed at a wide range of magnifications. In a previous study [11], we had already shown that one of these polymers, polyLM, displayed the same nanostructure reported for laminin arrays secreted by cells [12]. Moreover, we had described that polyLM and LM presented different morphologies in the range of tens of micrometers [8]. Nevertheless intermediary magnifications between these two ranges of sizes had never been assessed before. Using confocal, electronic and atomic force microscopy we filled in this gap and found that surprisingly polyLM presented similar structures independently of the magnification used to observe it. Since this property is a feature of fractals, we searched for a possible fractal dimension and confirmed that polyLM indeed corresponded to a fractal structure. Conversely, the second polymer, LM, was more heterogeneous and did not present a fractal nature.

Before addressing the biological significance of the present findings it is important to recapitulate the features of the polymers studied here. The term ordinary laminin (LM) is used to refer to clusters of laminin adsorbed onto a glass surface at a concentration below the critical concentration of 60–100 µg/ml, previously shown to induce solution polymerization at pH 7 [5]. In this condition the protein does not self-assemble in solution but it tends to form clusters as its concentration increases at the glass surface upon decantation/adsorption. Since laminin is used as a coating substrate for cell attachment at concentrations below the critical concentration (typically between 1 and 20 µg/ml), ordinary laminin can be considered as the standard form of the protein referred to in the literature. On the other hand, polylaminin (polyLM) is an artificial polymer generated upon pH acidification. It is formed independently of the protein concentration and it is not disrupted after increasing the pH to 7 [8]. It was initially described as a high molecular weight entity observed in solution by monitoring a spectroscopic parameter, namely static light scattering [6]. At a given medium and at a fixed wavelength, the intensity of light scattering is related to the size of the particles in solution, which allows for the use of this technique to follow the aggregation state of proteins in the presence or not of ligands or other interacting particles of biological interest [18]. Therefore, the 40-fold increase in light-scattering intensity observed upon transferring laminin from acidic to neutral buffer reflected an increase in the volume of protein particles in solution [6]. These particles were subsequently called “polymers”, instead of “aggregates”, which would suggest that they corresponded to clusters of denaturated protein. The term “polymer” was employed due to evidence that 1) the tertiary structure of laminin was preserved within the clustered particles [6]; 2) decanted/adsorbed particles formed biomimetic matrices both at the nano [11] and at the micrometer scales of size [8], and 3) key signaling properties of laminin were preserved and even augmented after the acid-induced assemblage, which was demonstrated mainly for neurons [8], but also for other cells types as glial [9] and thyroid cells [19].

The concept that the polymeric structure of proteins can influence their biological properties has gained increasing confirmation in recent years. In particular, laminin, a protein that occurs in the polymeric form in natural basement membranes, is known to have their signaling properties dependent on the establishment of the polymeric array [3], [4]. The interaction of laminin with other components of basement membranes, such as nidogens and/or the proteoglycan perlecan, is also influenced by polymerization, which is postulated to create new interacting sites at the nanoscale that did not exist in the individual molecule [20]. These studies however considered polymerization as having only two states (non-polymerized and polymerized). In other words, the polymeric state corresponded to a single entity, a supramolecular array in which the laminin trimers interacted with each other to form a sheet-like polymer anchored to the plasma membrane through cellular receptors. Such sheet-like polymer corresponds to the internal layer of basement membranes. However, there is evidence that certain tissues can produce other types of laminin structures. For instance, skeletal muscle fibers *in vitro* display membrane-bound deposits of laminin of two distinct morphologies [21], a reticular and a fibrillar one. These two morphologies were assigned to result from interactions with different cellular receptors, which, by presenting distinct regional distributions on the membrane, would lead to the formation of each laminin deposit. One complementary explanation, however, is that the morphologies of the two deposits at the micrometer scale would reflect specific molecular interactions at the nanometer range and therefore could involve interaction with the same receptor. In the nervous system, deposits of laminin appear with four different shapes throughout the development of the brain [22], [23]. In consonance with these *in vivo* findings, neurons isolated from lateral and medial regions of the midbrain have been shown to secrete either punctate or fibrillar laminin matrices, respectively [24]. Neurons from embryonic and neonatal brain cortex have also been shown to produce distinct laminin matrices, whereas one remained associated to the cell membrane, the other extended away from the cell surface, exhibiting the appearance of an array of tangled threads [13]. Finally, laminin deposits found in the neural stem cell niche, present two different morphologies described as puncta alternated with thin linear membranous structures anchored to blood vessels [25], [26]. We propose that the two laminin matrices observed in the present work reflect two alternative manners of protein assembly.

One important question to address is how this fractal would be generated and what would determine the self-assembly of laminin units into polyLM, LM or any other polymer. In a biological setting, the interaction with integrins and other laminin receptors would guide the process. As most of them bind to the long arm of laminin, it can be predicted that the simultaneous interaction of laminin trimers with membrane receptors will influence the formation of the type of polymerization. The ideal distance between two neighboring receptors to selectively favor interactions only among the short arms of laminin should correspond to the distance between the centers of the laminin molecules in the hexagonal network, *i.e.*, approximately 52 nm (Fig. 6). If the distance is such or shorter a flat polymer should be favored. On the other hand, as the spacing between receptors increased, other arrangements would be allowed. Interestingly, it has been shown that in fibroblasts and mesenchymal cells the ideal distance for signaling through integrins was in the range of 50–70 nm [27], [28].

In the case of a polymer generated in a cell-free system as polyLM the lack of cellular receptors demands an alternative explanation. By analyzing the distribution of surface charges, we have previously shown that the pH acidification necessary to trigger the formation of polyLM rends the distal portion of the long arm (fragments LG4 and LG5) completely positive [11]. We proposed that this would be the determinant for the prevention of the interaction between two long arms, which is the predominant laminin-laminin interaction in the absence of other molecules [5]. Therefore, polyLM could give rise to a hexagonal polymer mimicking the sheet-like polymer assembled on the cell surface even before adsorption to the coverslip. The previous report that polyLM increased the light scattering of the solution containing it, while LM in similar conditions did not, supports this notion [6]. The seed polymer already decants as a fractal structure within one hour of incubation and the complexity of the fractal increases as deposition proceeds (Fig. 8D). The height of the lowest structures found in polyLM and LM were around 60 nm and 120 nm, respectively (Fig. 5D, L). Interestingly, the former is the approximate size of the extended long arm of laminin (Fig. 6G). Such an extended conformation of individual laminin molecules has been previously detected by using electron microscopy after rotatory shadowing [5] and AFM [29]. Thus, it is likely that polyLM sediments as flat 2-D polymers in which only interactions among the short arms occur, as also suggested by the AFM measurements (Fig. 6D–F). On the other hand, in LM at least two layers of flat polymers mediated by interactions between long arms would be necessary to account for the 120 nm height observed in the lowest deposits.

In the present work we demonstrate that the protein laminin can give rise to a fractal structure. The observation that the supramolecular organization of a pure protein (polyLM) is fractal implies that the information contained ultimately within its primary sequence is sufficient to determine the morphology of larger structures that will spatially organize tissue compartments. This is particularly interesting because it correlates with the “fractal-like” organization of the niche for stem cells in the subventricular zone of the adult brain. In this case, a laminin-rich basement membrane, named “fractone”, has been proposed to orient the binding of proteoglycans, which, in turn, organize the distribution of the growth factors controlling the maintenance of the stem cell niche [26], [30], as shown to be the case for bFGF [31].

Although fractones have been described only in the central nervous system, it is well possible that a similar fractal-like extracellular matrix is present in other stem cell niches. Adult stem cell niches manifest under certain restricted microenvironments that host tissue-specific stem cells and regulate their physiology. They exhibit complex cytoarchitectures composed of stem cells, progenitor cells, supportive cells and a laminin-rich basement membrane. The basement membrane regulates cell division and differentiation within the niche due to several of its properties [32]. First, its molecular components interact with integrins to regulate the cytoskeletal assembly. It also harbors and controls the availability of growth factors and cytokines. A basement membrane provides an orienting surface for asymmetric cell division. Finally, it guides the traffic of progenitor cells within the niche. It is known that the assembly of basement membranes is initiated and dictated by laminin secretion and polymerization at the cell surface [33]. In addition, laminin is able to interact with integrins, to bind heparan sulfate proteoglycans, which, in turn, present soluble factors. It is thus very likely that laminin is the key component in basement membranes to play a regulatory role controlling the physiology of the stem cell niche. The importance of laminin for the maintenance of the stem cell niches has been demonstrated in several cases such as in pancreatic islets [34], [35], the germline niche [36], skeletal muscle [37] and the embryonic neocortical stem cell niche [38].

It is tempting to speculate that the fractal-like organization of stem cell niches can not only control the distribution of growth factors but also provide a physical constrain for progenitor cells during differentiation. The representation of a differentiation pathway as a tree, in which the stem corresponds to the stem cell and the branches, to progenitors is *per se* a fractal. In this scenario, the propagation of laminin polymerization throughout lager size scales could be a key step for the organization of multicellular organisms, which is in line with the observations that laminin-containing basement membranes are the first assembled extracellular matrix appearing during mammalian development and that laminin is present in virtually all metazoans [39].

## Supporting Information

Movie S1 **Animated view of the three-dimensional structure of polyLM.** The animation was generated from a series of confocal optical slices (the same shown in Figure 1A), using the software Imaris, version 7.2.3. (AVI)

Movie S2 **Animated view of the three-dimensional structure of LM.** The animation was generated from a series of confocal optical slices (the same shown in Figure 1B), using the software Imaris, version 7.2.3. (AVI)
